# Fear-Avoidance Behavior and Sickness Absence in Patients with Work-Related Musculoskeletal Disorders

**DOI:** 10.3390/medicina56120646

**Published:** 2020-11-26

**Authors:** Israel Macías-Toronjo, José L. Sánchez-Ramos, María J. Rojas-Ocaña, Esperanza Begoña García-Navarro

**Affiliations:** 1 Department of Rehabilitation, Huelva Fremap Hospital, 21007 Huelva, Spain; israel.macias123@alu.uhu.es; 2 Department of Nursing and Health Sciences, University of Huelva, 21007 Huelva, Spain; jsanchez@uhu.es; 3 Research Group ESEIS, Social Studies and Social Intervention, Center for Research in Contemporary Thought and Innovation for Development, (COIDESO), Department of Nursing and Health Sciences, University of Huelva, 21007 Huelva, Spain; bego.garcia@denf.uhu.es

**Keywords:** avoidance learning, neck pain, workplace, employment, fear, exercise, attitude, accidents, disability

## Abstract

(1) *Background and objectives*: The purpose of this work is to determine the association of fear-avoidance attitudes with sickness absence status, its duration and disability in a work accident context. (2) *Materials and Methods:* This is a descriptive observational design, conducting the study in two occupational insurance provider clinics with patients with nonspecific low back and neck pain during the study period. Clinical variables were the Fear Avoidance Questionnaire, Roland Morris Disability Questionnaire, Neck Disability Index, Numerical Pain Scale; sociodemographic variables were sex, age, occupational, educational level, sickness absence status, and duration in days of absence from work. Multiple logistic and linear regressions were used to explore the association between variables. (3) *Results:* Fear-avoidance behavior is related to sickness absence status (OR = 1.048, *p =* 0.007), and the physical activity dimension (OR = 1.098, *p* = 0.013) is more relevant than the work dimension (OR = 1.056, *p =* 0.028). The duration of sickness absence is related to higher values on the fear-avoidance behavior scale in its global dimension (b = 0.84, *p* = 0.003, r = 0.327), and the results of the physical activity dimension (B = 1.37, *p* = 0.035, r = 0.236) were more relevant than the work dimension (B = 1.21, *p* = 0.003, r = 0.324). Fear-avoidance behavior is related to disability in both dimensions (B = 0.912, *p* ˂ 0.001, r = 0.505). (4) *Conclusions*: Fear-avoidance behaviors may influence the typification of sickness absence status, its duration both in its physical activity and work dimension, and its disability reported with higher values than in other healthcare contexts.

## 1. Introduction

Fear-avoidance behaviors are associated in an important way to the evolution and transition towards chronicity of musculoskeletal pain disorders [1,2]. The fear avoidance model focuses on patients’ beliefs about disease, movement, and pain by creating myths related to erroneous thoughts about the nociceptive experience. According to this model, there would be an asynchrony between the natural pathological process and the clinical manifestation referred by the patient [3]. The pain referred would be exaggerated and not in accordance with the normal physiological process of the disorder. A central element of this model is the interpretation that the patient makes of an nociceptive experience as the origin of an important physical damage over which the patient has no control [3]. The avoidance and pain hypervigilance behaviors are based on catastrophic thoughts that activate limiting attitudes that, in turn, amplify the disability and pain [2]. As a result, catastrophic thoughts are related to fear of movement with worse results in terms of prognosis in therapeutic outcomes [4,5,6].

The working environment could be responsible for at least 37% of cases of low back pain (LBP) worldwide [7,8]. Workers exposed to forced postures, heavy lifting, or physically demanding jobs appear to be more likely to experience episodes of neck pain (NP) and LBP [9,10]. In addition, low levels of social coverage, job dissatisfaction, or stress levels caused by emergency situations could be associated with the development of musculoskeletal disorders [7,11]. 

High levels of fear-avoidance behaviors are associated with limitation of activity and greater levels of disability [12] and could condition recovery in processes of musculoskeletal disorders. In addition, fear-avoidance beliefs appear to be a predisposing factor towards long-term disorder processes, and their predictive value in acute problems is uncertain [13]. In this sense, the few studied which have focused on studying the association between fear-avoidance attitudes and time to return to work, in an occupational context, have questionable results [13,14,15], although the evaluation of this type of beliefs seems to have a positive result for determining the prognosis of patients with musculoskeletal disorders [14].

Different authors have insisted on the identification of psychosocial risk factors in the management of patients with musculoskeletal disorders [16,17,18]. Fear-avoidance behaviors and their influence on sickness absence and return to work have been scarcely studied in an occupational context. Musculoskeletal pain has been evaluated from the point of view of primary care within public health care [19,20,21], but more studies in an occupational health insurance provider context are necessary, taking into account that, in Spain, occupational disease is specifically managed by these institutions. In many European countries, these occupational health systems, similar to their Spanish counterparts, are responsible for managing occupational accidents [22], with a varied structure within the European Union [23]. Currently, Europe, Australia, Canada, USA, and others consider this system to be an essential part of the management of accidents and professional illness [23,24]. In Spain, occupational health insurance providers are non-profit organizations that manage health care and economic coverage in the process of an occupational accident or occupational disease. In this sense, these organizations have specific and earlier resources (specialized medicine, imaging tests, or rehabilitative treatment) as compared with public health services.

Considering that the presence of fear-avoidance attitudes may be associated with the evolution of low back and neck pain symptoms, the objective of this work was to determine the relationship of these variables as predictors of sickness absence status and its duration in an occupational health insurance provider context. Therefore, the aim of this article is to describe the association between fear-avoidance behaviors as psychosocial factors in patients with occupational low back pain (LBP) and neck pain (NP) and their influence on absenteeism in an occupational health insurance provider context. This analysis is justified by the need to identify these non-strictly clinical variables for prevalent disorders such as low back and neck pain, in order that they can be observed and taken into account in guidelines and multidisciplinary therapeutic approaches at an occupational context.

## 2. Materials and Methods 

### 2.1. Type of Study

This is a descriptive observational study conducted at the Clinical Health Service of an occupational insurance provider.

### 2.2. Subjects

All the subjects who presented to an occupational health clinic with a diagnosis of nonspecific low back and neck pain due to a work accident between 1 June 2018 and 31 December 2019 and met the inclusion criteria were included in this study. Nonspecific pain is considered to be pain that is not caused by fractures, direct trauma, or systemic disease and where there is no proven root compression amenable to surgical treatment [25]. A work-related accident is any bodily injury that the employed worker suffers during the time, or as a consequence, of work. The target population was the entire group of patients who met the inclusion criteria. All patients (*n* = 129) who met this inclusion criteria were consecutively included.

The following inclusion criteria were applied:

1. Work-related nonspecific LBP and NP attending to an occupational health clinic;
2. Age between 18–65 years old;
3. Understanding the language, informed and signed consent.

The following exclusion criteria were applied:

1. Back pain or neck pain related to infection, cancer, fracture, visceral disease, spondylarthrosis, extruded disc herniation, or cauda equina syndrome;
2. Previous treatment for spinal pain;
3. Previous surgical intervention, commuting accident, common illness, or occupational disease;
4. Cognitive impairment.

The injured patients were attended by the occupational insurance medical service the same day as the work accident and, after diagnosis and typification as a work accident, it was noted if the patients were on sick leave or if they could reconcile their disorders with work activity. At the end of their visit with the physician, each patient who met the eligibility criteria was informed of the study objectives and was asked to participate. All patients included in the study signed the relevant informed consent form. The participants knew that the information collected was confidential and anonymous. 

**Figure 1:**
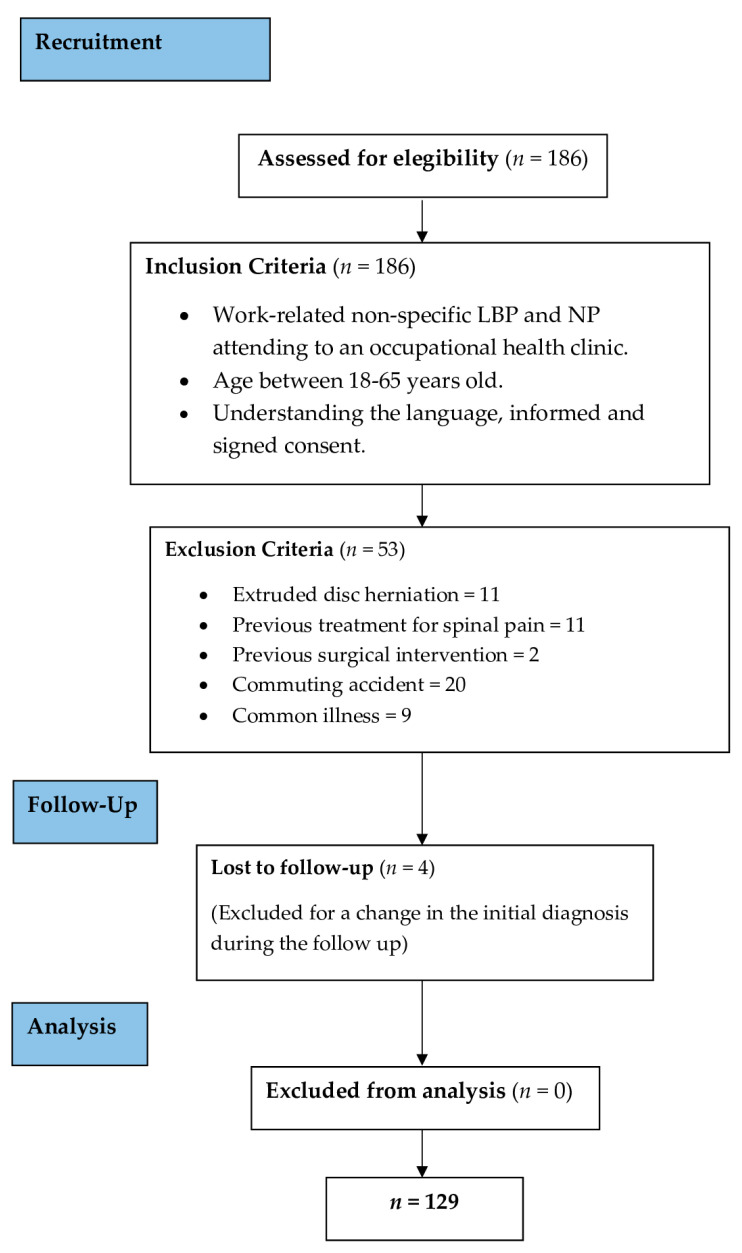
Recruitment process diagram.

On the first day of attendance, the participants were interviewed by the physiotherapist in charge and sociodemographic information was collected, the content of the questionnaires was explained, and the questionnaires were completed only once, prior to any other therapeutic intervention. The duration of sick leave was measured by the days off from the day of the accident at work until the patients returned to work. Any re-injury or setback of the same LBP and NP disorder after the six months after the day of return to work were considered to be part of the same process of sickness absence and therefore were recorded as the same sickness absence. Any change in the initial diagnosis of nonspecific low back or neck pain of any of the participants throughout the follow-up process would cause them to not meet the inclusion criteria and be excluded from it. The recruitment process is shown in Figure 1.

The study was authorized by the Fremap Mutual Ethics Committee (code number FREMAP-2200631-Z) and followed the ethical principles for medical research in human beings according to the Declaration of Helsinki and the protection of data and guarantees of digital rights according to organic law March 2018 of 5 December 2018.

### 2.3. Study Variables

#### 2.3.1. Fear-Avoidance Questionnaire (FABQ)

Fear-avoidance behaviors about work are closely related to sickness absence in musculoskeletal disorders processes [26]. The questionnaire reflects how physical and work activity influence the nociceptive experience [27]. It consists of two parts, one on fear-avoidance beliefs during work activity (Cronbach’s alpha = 0.88) and another on physical activity (Cronbach’s alpha = 0.77). The FABQ contains 16 items, 5 for a physical activity subscale (with a maximum score of 24), and 11 for the work subscale (with a maximum score of 42). Each item is answered on a 7-point Likert scale where 0 is totally disagree and 6 totally agrees. The Spanish version of the FABQ has good comprehensibility, consistency, and reliability, with the full version being as valid as both subscales, as well as being easier to score and analyze, thus, facilitating its use in clinical practice [27]. In this study, the full scale (Cronbach’s alpha = 0.84) and both dimensions were used separately.

#### 2.3.2. Roland Morris Disability Questionnaire (RMQ)

Disability was measured using the Roland Morris Questionnaire instrument. It is one of the world’s best validated and globally used scales to measure LBP disability. It consists of 24 items related specifically to daily physical activities likely to be affected by LBP scoring between 0 (no disability) and 24 (maximum disability). A reliable Spanish version has been validated to evaluate disability in patients with LBP [28], with an intraclass correlation of 0.87, good concurrent and construct validity, and a high internal consistency. Disability is expressed in absolute values. 

#### 2.3.3. Neck Disability Index Questionnaire (NDI)

Neck disability was measured with the Neck Disability Index Questionnaire. This 10-item questionnaire reflects limitations in activities of daily living of NP patients. It has 6 possible answers for each item scored from 0 to 5. It is a validated and reliable Spanish version to measure disability in patients with NP [29]. Neck pain disability is expressed in absolute values.

#### 2.3.4. Numerical Pain Scale

The Numerical Pain Scale, an 11-item scale, in which 0 indicates an absence of pain while 10 represents the worst imaginable pain, was used to measure pain intensity [30].

#### 2.3.5. Socio-Demographic Variables

Socio-demographic variables were collected on sex, age, occupational and educational level, as well as the following clinical variables: location, sickness absences status, and its duration.

For occupational level variable, the National Classification of Occupations 2011 (NCO-11) prepared by the Spanish National Statistics Institute (INE) was taken as a reference in the classification according to the level of skills. It consists of four skill levels [31]. The subjects were grouped into primary education, secondary education, pre-university education, and university education for the educational level variable.

### 2.4. Statistical Analysis

Firstly, a descriptive analysis of the variables studied was carried out. Means and standard deviations (SD) were incorporated for continuous variables and absolute and relative frequencies for categorical variables. To analyze the relationship between variables, a bivariate analysis of the variables associated with the sickness absence status, the duration of sickness absence and disability was carried out. The relationship of socio-demographic variables (sex, education, occupation, and location) with sickness absence status was checked using the Chi-square statistic, once the conditions of application were verified. The level of professional competence was recoded, grouping the only case of Level 4 with those of Level 3. The means of the clinical variables and age were compared using Student’s *t*-test to see the differences between patients who were on sickness absence status and those who were not, after checking the absence of deviations by means of a detrended Q-Q plot and variance homogeneity. A linear regression model was used, controlling for occupation and educational level variables, to evaluate the association of fear-avoidance beliefs with the duration of sickness absence and the degree of disability. Multiple logistic regression was used, controlling for socio-demographic variables, to explore the association of the variables fear avoidance behaviors and pain intensity with the need for sickness absence. Using multiple linear regression, controlling for the socio-demographic variables, the association of the variables fear avoidance behaviors with the duration of sickness absence and the degree of disability was explored.

The Statistical Package for the Social Sciences (SPSS) software program was used for the statistical analysis in this work.

## 3. Results

This study included 129 subjects in which 71 were men (55%) and 58 women (45%) (CI 95% 0.52–2.17). Patients with low back pain were the majority (*n* = 88, 68.2%) versus neck pain (*n* = 41, 31.8%). Subjects on sickness absence (*n* = 81 vs. *n* = 48) status were the majority (62.8% vs. 37.2%) (CI 95% 0.23–1.05). The mean values of the age variable were 40.1 (standard deviation = 9.43) years. Mean duration of sickness absence was 23.74 (SD = 30.27) days. The levels of disability were higher in the group with low back pain (46.64%) than in the group of patients with neck pain (42.67%). The average of the Fear-Avoidance Behavior Questionnaire in its global dimension was 45.09 (SD = 13.15), in the physical activity dimension it was 18.03 (SD = 5.93), and in its work dimension it was 27.05 (9.15). The Numeric Pain Scale mean score was 7.02 (1.83).

### 3.1. Sickness Absence

The relationship between presenting fear-avoidance behavior and sickness absence is described in Table 1. Subjects on sickness absence presented a higher mean score on the fear-avoidance behavior scale (46.63 (11.80)) than those not on sickness absence (39.10 (13.24)) (*p* < 0.001). Subjects on sickness absence presented a higher mean score on the fear-avoidance scale for work (29.19 (8.13)) and physical activity (19.44 (5.21)) than those not on sickness absence status (23.46 (9.71)) (16.65 (6.35)) (*p* < 0.001). In the results presented, fear-avoidance behavior is related to sickness absence in its global version (OR = 1.05, *p* = 0.007), the relationship with the physical activity dimension (OR = 1.10, *p* = 0.013) was higher than with the work dimension (OR = 1.06, *p* = 0.028) (Table 2).

### 3.2. Duration of Sickness Absence

No relationship between socio-demographic variables and the duration of sickness absence was found. Regarding the clinical variables, the duration of work absence rises by 0.84 days for each unit of increase in the fear-avoidance behavior scale on its global dimension (B = 0.84, *p* = 0.003, r = 0.33), and the relationship with the physical activity dimension (B = 1.37, *p* = 0.035, r = 0.24) was superior as compared with the work dimension (B = 1.21, *p* = 0.003, r = 0.32) (Table 3).

### 3.3. Disability

Regarding the association between fear-avoidance attitudes and disability, this variable increases 0.91 units for each point on the global fear-avoidance attitudes scale (B = 0.91, *p* ˂ 0.001, r = 0.50), and the scale on physical activity was superior (B = 1.70, *p* ˂ 0.001, r = 0.41) as compared with the work scale (B = 1.1, *p* ˂ 0.001, r = 0.42). The relationship between pain intensity and disability shows statistical significance (B = 4.36, *p* ˂ 0.001, r = 0.33). In multiple linear regression among fear-avoidance attitudes, intensity of pain, and disability, the scale of fear-avoidance attitudes remains stable in both groups of subjects for sickness absence status (B = 0.81, *p* ˂ 0.001, r = 0.44) and in the group of subjects who remain active at work (B = 0.81, *p* ˂ 0.001, r = 0.44) (Table 4).

## 4. Discussion

### 4.1. Sickness Absence and Duration of Absence

The results presented indicate an association of fear-avoidance behavior with sickness absence status and with the time to return to work in patients with work-related LBP and NP. This association is reflected both in attitudes related to physical activity and in those related to the work dimension. In this analysis, education and occupation variables have been considered to be confounding variables, and therefore controlled in the statistical process. 

The ability of the fear-avoidance questionnaire to predict sickness absence status and time to return to work has previously been shown to be questionable [32]. However, other authors have pointed out that high levels of fear-avoidance attitudes in the work dimension were related to long term sickness absence and no improvement in disability and pain in patients with LBP [33]. The results presented, in this study, are based on data collected at the beginning of the sickness absence process and on the follow-up of the duration of the absence. In this sense, these authors worked with a sample of chronic patients, while the results obtained in this project were collected from a follow-up of acute, subacute, or chronic patients until the day of return to work. Therefore, the control of the subjects in the sample presented in this work was done from the first day of the work accident, which would make the follow up of the measure stricter without any time period from the accident until the patient started to be controlled. 

In the analysis presented in this project, the two dimensions are related to both the sickness absence status and the duration of sickness absence, with the physical activity dimension having a stronger association. Grotle et al., in a follow-up study at 3, 6, 9, and 12 months, reported that chronic pain patients presented significantly higher values of fear-avoidance behaviors in the work dimension than those in acute pain [34]. In this regard, these authors [34] described that the results of the fear-avoidance questionnaire in the physical activity dimension gave higher initial results that were reduced during the first month of follow-up, which could explain why the patients in the sample presented higher levels of fear-avoidance attitudes in the physical activity dimension than in the work dimension. IN addition, it is the occupational health insurance provider that, in addition to managing health care, manages the economic compensations derived from sickness absence. In a sample of subjects in which occupational primary sector and nonpermanent jobs prevail, the answers referring to their own capacity to work or when they would expect to return to work included in the questionnaire in its work dimension, could be biased by an economic issue. Thus, the question referring to the economic compensation received for being in a sickness absence status (item eight in the questionnaire on fear-avoidance behavior) was, on many occasions, a reason for consultation by the workers on the obligatory nature of the response. In the same line, the fear-avoidance behavior questionnaire has been debated by some authors as a one-dimensional instrument capable of measuring fears and avoidance attitudes towards pain [35]. These authors proposed, in a detailed analysis of each of the items in the questionnaire, that it indicated more expectations for returning to work than actual fears and avoidance attitudes towards patient work activity. This, together with the bias proposed in the context of sickness absence/economic compensation, could question the use of this work dimension of the questionnaire in an occupational health insurance provider context. In any case, the data on both sickness absence status and its duration are related to both dimensions of fear-avoidance behavior, although in an occupational context, a greater association with the work dimension could be expected.

Storm et al. related high scores in their work dimension to the ability to work in a short four-week longitudinal study and considered it to be an important tool when assessing work capacity in patients with back pain [36]. Along the same lines, Jay et al., in a study that investigated the relationship among fear-avoidance variables with the time to return to work in patients with musculoskeletal disorders (lumbar, cervical/shoulder, and arm/hand pain), indicated that these psychosocial variables were related to absenteeism from work [37], something in line with the results presented in this work. In our case, the sample consisted exclusively of subjects who had suffered a work-related accident, while the sample of the authors mentioned above were on subjects on work and non-work-related sickness absence in a mixed population sample. In this sense, this work is novel by the fact that it relates work-related sickness absence with this type of attitude.

The values of the fear-avoidance behavior obtained are similar to other studies both in acute [38,39] and in chronic pain patients [40]. These data are also consistent with other observational studies in the same line [19,33,41,42], giving meaning to the fear avoidance model, according to which the perception of pain in the short term could lead to movement avoidance behavior, reinforcing this attitude in the long term and providing negative characteristics to movement after a work accident [42]. 

Both dimensions of the fear-avoidance behavior scale are related to the duration of sickness absence with a higher relevance than in other studies [33]. Thus, Kovacs et al., in a primary care study in the Spanish Health System, although they did not report a relationship between fear-avoidance behavior and disability, they did find a relationship between the duration of sickness absence in patients with low back pain during the year following the injury in the context of chronic pain [19]. In general, fear-avoidance behavior values are higher in workers who remain sedentary than in those who perform some activity [19,43], which is in line with the results of this work on a global scale and in both dimensions separately but, in this case, in a much more intense association [40]. The results obtained could be influenced by such an early intervention and this, in turn, could be related to the fact that the observed association between fear-avoidance behaviors and sickness absence and their duration was so strong. Be that as it may, the work environment seems to mediate this association in some way.

### 4.2. Disability

The results presented show a strong association between fear-avoidance behavior, in both dimensions, and declared disability in first attendance. In this same sense, Trinderup et al. related this type of attitude towards pain and disability in patients with chronic LBP [33]. In cross-sectional studies, this association with fear-avoidance behavior has been confirmed to be the main predictor of LBP disability in a study carried out in a primary care system [44]. In Spain, the influence of fear-avoidance behavior on disability in primary care has been previously studied, showing no association [19], or being clinically irrelevant [21]. Our study, in multivariate analysis, with the control of confounding variables (education and occupation) and including the intensity of pain declared in first assistance, does show a correlation between fear-avoidance behavior and pain intensity with disability both in patients on sickness absence as in the work active patients. Again, contrary to what might be expected, attitudes related to physical activity are more related to disability than occupational ones, and items related to return to work and economic compensations could influence the results at this level. 

In the same line of the presented results, intensity of pain has also been pointed out as a predictor of disability in prospective studies [45] and likewise, the information collected was done the same day of the accident. This early intervention could determine the high levels of pain reported.

Thus, the reported disability would be related, according to our results, to fear-avoidance behavior and the amount of pain declared regardless of the work status of the subjects.

In this study, the subjects were recruited on the day of the work accident and followed up until the day of return to work. In this sense, the obtained results and interpretations could be influenced by this very early recruitment. Regarding the limitations of this work, other aspects of a psychological nature such as anxiety and depression have also been shown to intervene in patients’ return to work [46,47,48]. In this work, this specific aspect has not been controlled and could be a confounding factor to our results. Furthermore, the methodological design used would not allow us to establish causal relationships among the variables studied. Although the follow-up was carried out until the day of return to work, we would recommend longitudinal studies with larger sample sizes and more heterogeneous work groups.

## 5. Conclusions

Both dimensions of the fear-avoidance behavior questionnaire are related in a work-related health insurance provider context to the sickness absence status, its duration, and disability reported with higher values than in other health areas, and fear-avoidance behavior is lower in subjects who remain active at work.

Fear-avoidance behavior must be taken into account for the assessment and follow-up of these patients with the aim of reducing the time to return to work, work absenteeism, and achieving better results in clinical interventions.

## Figures and Tables

**Table 1:** Mean values for the different clinical variables as a function of the presence (yes) or absence (no) of sickness absence.

Sickness Absence	Yes			No			‡ *p*
	*n*	Mean	SD	*n*	Mean	SD	
Fear-avoidance behavior	81	48.63	11.80	48	39.10	13.24	<0.0001
Fear-avoidance Work	81	29.19	8.13	48	23.46	9.71	<0.0001
Fear-avoidance physical activity	81	19.44	5.21	48	15.65	6.35	<0.0001
Intensity of pain	81	7.23	1.55	48	6.69	2.19	0.101

‡ t-Student.

**Table 2:** Multiple logistic regression model. Influence of fear-avoidance behavior on the sickness absence variable, controlled by education and occupation.

Multiple logistic regression model: influence of fear-avoidance attitudes on sickness absence, controlled by education and occupation.			
**Sickness absence**	***p***	**Odds Ratio**	**CI 95% Odds Ratio**
Fear avoidance	0.007	1.05	[1.01–1.08]
Education	0.090		
Primary/University	0.591	2.12	[0.14–32.59]
Secondary/University	0.905	0.85	[0.05–13.21]
Pre-University/University	0.862	1.26	[0.10–16.51]
Occupation	0.299		
Level 1/Level 3	0.291	3.86	[0.31–47.52]
Level 2/Level 3	0.844	1.32	[0.08–22.04]
2. Multiple logistic regression model: influence of fear-avoidance physical activity behavior on sickness absence, controlled by education and occupation			
**Sickness absence**	***p***	**Odds Ratio**	**CI 95% Odds Ratio**
Fear avoidance PA *	0.013	1.10	[1.02–1.18]
Education	0.275		
Primary/University	0.638	1.91	[0.13–27.96]
Secondary/University	0.836	0.75	[0.05–11.22]
Pre-University/University	0.940	1.10	[0.09–13.97]
Occupation	0.208		
Level 1/Level 3	0.193	5.18	[0.43–61.57]
Level 2/Level 3	0.730	1.64	[0.10–26.90]
3. Multiple logistic regression model: influence of fear-avoidance work behavior on sickness absence, controlled by education and occupation.			
**Sickness absence**	***p***	**Odds Ratio**	**CI 95% Odds Ratio**
Fear-avoidance work	0.028	1.06	[1.01–1.11]
Education	0.290		
Primary/University	0.534	2.35	[0.16–34.97]
Secondary/University	0.977	0.96	[0.06–14.46]
Pre-University/University	0.841	1.30	[0.10–16.67]
Occupation	0.280		
Level 1/Level 3	0.269	4.08	[0.34–49.60]
Level 2/Level 3	0.807	1.42	[0.09–23.32]

* Fear-avoidance physical activity.

**Table 3:** Multiple linear regression of the duration variable with clinical variables controlling for occupation and education variables.

Duration of Sickness Absence	b	95% CI (b)	r	*p*
Fear-avoidance behavior	0.84	[0.29–1.38]	0.33	0.003
Fear-avoidance work	1.21	[0.41–2.00]	0.32	0.003
Fear-avoidance physical activity	1.37	[0.10–2.64]	0.24	0.035
Pain intensity	2.78	[−1.58–7.15]	0.14	0.208

**Table 4:** Multiple linear regression of the disability variable with clinical variables individually controlled by the confounding variables education and occupation (initial and final model).

(a) Multiple linear regression of the disability variable with clinical variables individually controlled by the confounding variables education and occupation (initial model).				
**Disability**	**B**	**CI 95% Odds Ratio**	**r**	***p***
Fear avoidance	0.91	[0.61–1.22]	0.50	<0.0001
Fear-avoidance work	1.10	[0.65–1.55]	0.42	<0.0001
Fear-avoidance physical activity	1.66	[0.99–2.34]	0.41	<0.0001
Pain intensity	4.36	[2.18–6.54]	0.33	<0.0001
(b) Multiple linear regression of the disability variable with clinical variables individually controlled by the confounding variables education and occupation (final model).				
**Disability**	**B**	**CI 95% Odds Ratio**	**r**	***p***
Fear avoidance	0.81	[0.51–1.11]	0.44	<0.0001
Pain intensity	3.19	[1.17–5.22]	0.24	0.002
(c) Multiple linear regression of the disability variable with clinical variables in the context of sickness absence (final model).				
**Disability**	**B**	**CI 95% Odds Ratio**	**r**	***p***
Fear avoidance	0.88	[0.50–1.26]	0.44	<0.0001
Pain intensity	4.63	[1.76–7.51]	0.30	0.002
